# Author Response: Effect of Depth-Dependent Integrated Visual Field on Vision-Related Quality of Life in Glaucoma

**DOI:** 10.1167/tvst.13.12.2

**Published:** 2024-12-02

**Authors:** Andrew Turpin, Allison M. McKendrick

**Affiliations:** 1 Lions Eye Institute, Perth, Australia; 2 Curtin University, Perth, Australia; 3 University of Western Australia, Perth, Australia; 4 University of Melbourne, Parkville, Australia. e-mail: andrew.turpin@lei.org.au

**Keywords:** glaucoma, binocular visual field, quality of life

We were very pleased to see that Gazanchian and Jansonius have made use of the method described in our previous publication, “A Depth-Dependent Integrated VF Simulation for Analysis and Visualization of Glaucomatous VF Defects,”^1^ and we would like to point interested readers to the new location for the online version of the R package: https://binovisualfields.lei.org.au/binovisualfields.

Gazanchian and Jansonius estimated binocular integrated visual fields at distances of 100 cm and 25 cm (in both cases, the observer was fixating at 60 cm). These distances represent many daily activities in peripersonal space (for example, reaching for a coffee cup on your desk while still watching your laptop screen). The validated questionnaires used by the authors are not specifically designed as sensitive instruments for self-reported difficulty on these types of tasks. Prior literature demonstrates that glaucoma can impact both the precision and time taken to successfully complete tasks with a visuomotor component.^2^^–^^5^ Indeed, part of our original motivation for devising the method to quantify depth-dependent integrated visual fields was the observation that people with visual field loss are slower at reaching, grasping, and pointing tasks than age-matched controls.^3^^,^^4^ We suggest that further work should be conducted using such methods to determine whether the size of “volume scotoma” impacts the accuracy or speed of task performance requiring depth judgments in peripersonal space.
